# Iron Deficiency Anemia in Pediatric Gastroesophageal Reflux Disease

**DOI:** 10.3390/diagnostics13010063

**Published:** 2022-12-26

**Authors:** Vasile Valeriu Lupu, Ingrith Miron, Ana Maria Laura Buga, Cristina Gavrilovici, Elena Tarca, Anca Adam Raileanu, Iuliana Magdalena Starcea, Andrei Tudor Cernomaz, Adriana Mocanu, Ancuta Lupu

**Affiliations:** 1 Pediatrics Department, “Grigore T. Popa” University of Medicine and Pharmacy, 700115 Iasi, Romania; 2 Department of Surgery II—Pediatric Surgery, “Grigore T. Popa” University of Medicine and Pharmacy, 700115 Iasi, Romania; 3 3rd Medical Department, “Grigore T. Popa” University of Medicine and Pharmacy, 700115 Iasi, Romania

**Keywords:** gastroesophageal reflux, anemia, esophagitis, children

## Abstract

(1) Background: Gastroesophageal reflux disease (GERD) can cause several complications as a result of the acidic pH over various cellular structures, which have been demonstrated and evaluated over time. Anemia can occur due to iron loss from erosions caused by acidic gastric content. In children, anemia has consequences that, in time, can affect their normal development. This study evaluates the presence of anemia as a result of pediatric gastroesophageal reflux disease. (2) Methods: 172 children were diagnosed with gastroesophageal reflux in the gastroenterology department of a regional children’s hospital in northeast Romania by esophageal pH-metry and they were evaluated for presence of anemia. (3) Results: 23 patients with GERD from the studied group also had anemia, showing a moderate correlation (r = −0.35, *p* = 0.025, 95% confidence interval) and lower levels of serum iron were found in cases with GERD, with statistical significance (F = 8.46, *p* = 0.012, 95% confidence interval). (4) Conclusions: The results of our study suggest that there is a relationship between anemia or iron deficiency and gastroesophageal reflux due to reflux esophagitis in children, which needs to be further studied in larger groups to assess the repercussions on children’s development.

## 1. Introduction

Gastroesophageal reflux represents the retrograde movement of gastric content into the esophagus. This phenomenon is physiological and can occur several times a day, for all age groups [1].

**Figure 1:**
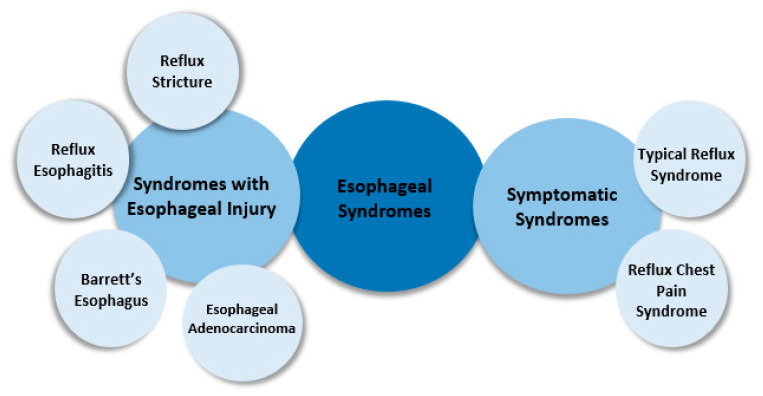
Esophageal syndromes of GERD [2].

**Figure 2:**
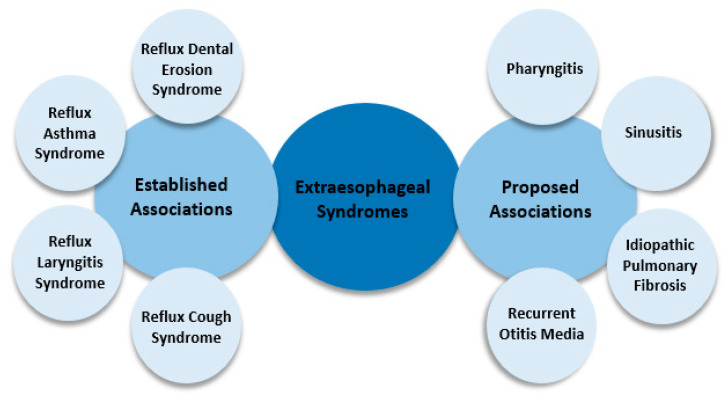
Extraesophageal syndromes of GERD [2].

According to the Montreal consensus in 2006, when gastroesophageal reflux leads to symptoms that interfere with a person’s well-being and/or the occurrence of complications, it is referred to as gastroesophageal reflux disease (GERD). The complications included in the definition of gastroesophageal reflux disease can be grouped into esophageal and extraesophageal syndromes, as presented in Figure 1 and Figure 2 [2].

In pediatric gastroesophageal reflux disease, the symptomatology varies according to age and is non-specific. If for older children and adolescents this is similar to those present in the adult patient, in infants, the manifestations are often general or superimposed on the onset of other diseases (regurgitations, arching, feeding refusal, weight loss or weight stagnation, agitation, irritability, inconsolable crying, etc.) [3].

The diagnosis of GERD is established both clinically, based on symptoms and signs, and paraclinical, by identifying lesions caused by acidic pH through upper digestive endoscopy (esophagitis of various degrees, Barrett’s esophagus, or adenocarcinoma) or by determining the presence of low pH in the esophagus by esophageal pH-metry. Continuous esophageal pH-metry was introduced in the 1990s and represented the gold standard for the diagnosis of GERD until the introduction of impedance pH-metry. Technological advances led to the possibility of performing wireless esophageal pH-metry, by means of a capsule that attaches to the esophageal mucosa and detaches after 48 h, as an alternative to classical monitoring, but at the moment, this technique is mainly used in the United States of America [4].

The prolonged action of acidic pH on the esophageal epithelial cells can lead to various important consequences, from feeding intolerance and malnutrition in infants to respiratory pathology in those predisposed (i.e., children with Down syndrome or neuromuscular diseases) and, in extreme situations, to Barrett’s esophagus at older ages. Last, but not least, iron loss from bleedings along the esophagus wall can determine iron deficiency or hypochromic hyposideremic anemia in the long term [5].

Iron deficiency anemia (IDA) is the most frequent types of anemia and for pediatric patients has an incidence between 39% and 48.1% in developing countries and between 5.9% and 20%, depending on age, in developed countries [6]. The main mechanism that leads to iron deficiency anemia is low nutritional sources of iron from diet both in industrialized and developing countries, the prevalence for iron deficiency being four times higher in developing countries as opposed to developed ones. In the long run, anemia has a negative impact on growth, cognitive function, and behavior of children, but also in the case of adults, it produces unwanted effects such as physical and mental asthenia, restless leg syndrome, for pregnant women it can cause premature birth, low birth weight children, or even mortality for both mother and fetus [7]. Beside low dietary intake being the main cause of iron deficiency, there are other sources that ultimately lead to iron deficiency anemia, as summarized in Table 1 [8].

Since in our daily clinical practice we encountered many cases of anemia in which other etiologies were excluded through anamnesis and various paraclinical explorations, and at the same time we also observed the large number of patients with gastroesophageal reflux, we proposed to analyze in this study the relationship between these two pathologies.

## 2. Materials and Methods

We conducted a retrospective study over a period of 5 years, on 234 patients hospitalized in the gastroenterology department of a regional children’s hospital in north–east Romania with suspicion of GERD. The diagnosis of gastroesophageal reflux was established following the results of esophageal pH-metry and that of iron deficiency anemia through the measurement of hemoglobin, hematocrit, mean cellular volume, mean cellular hemoglobin concentration, and serum ferritin.

Informed consent was obtained from all patients’ carers, and the “St. Mary” Children Emergency Hospital Ethics Committee’s approval was obtained.

Indications for esophageal pH-metry were the presence of GERD symptoms and signs: cough unresponsive to treatment, nocturnal cough, odynophagia, frequent regurgitation or vomiting without other cause, prolonged crying in infants, unsatisfactory weight gain in infants and small children.

The exclusion criteria from the study were: treatment with antacid medication in the last 3 months, treatment with non-steroidal anti-inflammatory drugs or aspirin, treatment for Helicobacter pylori eradication, gastrointestinal bleeding diagnosed through endoscopy, esophageal strictures or esophagitis in the context of systemic diseases, personal history of surgery on the esophagus or stomach [9], and other causes of iron deficiency anemia (excluded through detailed medical history, physical examination).

To determine the pH, the equipment used was Medtronic Digitrapper^R^ pH 100, SN 37660, the catheters used were Zinetics 24 and ComforTec by Sandhill. Software used for recording the measurements was Polygram.Net^TM^ pH. The values were recorded continuously for 24 h through a probe placed 5 cm above the lower esophageal sphincter; the difference between the physiological and pathological reflux periods were made by the pH values below 4. The diagnosis of GERD was established according to the obtained values of the Boix-Ochoa score, a value lower than 11.99 being considered normal, according to the literature [9,10].

The diagnosis of anemia was established based on the low values for the age range for hemoglobin and hematocrit (see Table 2). We also quantified mean cellular volume (MCV), mean cellular hemoglobin (MCH), mean cellular hemoglobin concentration (MCHC), and serum iron values and compared them to the normal values (see Table 3 and Table 4).

Software used for data processing was IBM SPSS Statistics 20 and the Pearson parametric correlation was used for correlation analysis. Furthermore, the correlation coefficients were calculated for a confidence interval of 95%.

## 3. Results

Among the 234 children included in the study, 32 patients had anemia and 172 patients were identified with gastroesophageal reflux disease through a positive Boix-Ochoa score (N < 11.99). From the 32 patients with anemia, 23 were associated with GERD, a higher number than those without GERD (F = 7.86, *p* = 0.0173, 95% confidence interval). The statistical data analysis shows a moderate correlation between anemia and GERD (r = −0.35, *p* = 0.025, 95% confidence interval). At least two erythrocyte parameters are significantly affected, namely, MCH and MCV, which translates into interpretating terms indicating a tendency towards deficiency anemia (Table 5 and Table 6). The decrease in MCH is obvious and meets the conditions of statistical significance even when statistical correlation tests are applied. This aspect actually confirms that these children have a marked tendency to become anemic during the evolution of the disease and the erythrocyte parameters can be the first detectable changes with indicative value.

The serum iron level was investigated in patients who had modified erythrocyte parameters even if the hemoglobin and hematocrit values were normal. We identified lower levels of serum iron in cases with GERD, with statistical significance (F = 8.46, *p* = 0.012, 95% confidence interval). We must emphasize the fact that this serum parameter also correlates very well with the imminence of anemia as suggested by the other parameters. The values found in patients with GERD are much lower compared to the control group and compared to the normal inferior limit of sideremia (Table 7), suggesting the fact that the origin of the tendency towards anemia is in fact the iron deficiency achieved both by chronic losses and by affecting the intake.

Gender distribution and the area of living for the patients in the studied group are summarized in Table 8.

## 4. Discussion

From a pathophysiological point of view, in infants, gastroesophageal reflux is due to the immaturity of the smooth muscle fibers of the lower esophageal sphincter, which allows the passage of the gastric contents back into the esophagus, leading to the destruction of the esophageal epithelial cells through its acidic pH, with inflammation and irritation [12]. Furthermore, there is a lower resting pressure in the lower esophageal sphincter of the infants compared to that of the adult, starting at 3.8 mmHg for premature infants born at 27 weeks of gestation, 12.2 mmHg for the ones born at 35 weeks of gestation, and reaching 18 mmHg for full-term infants versus 19 up to 28 mmHg for an adult [13]. At the same time, the exclusively liquid diet and prolonged periods in a horizontal position also contribute to the occurrence of GERD in infants under the age of 6 months [12]. Also, there are more cases of GER at younger ages due to another series of peculiarities at that stage in life: the esophageal capacity is 5–10 mL, unlike that of an adult that has approximately 180 mL, the intra-abdominal part of the esophagus is shorter than in the adult, which is about 3 cm, and last, but not least, infants ingest a volume of food per kilogram of body five to eight times greater than adults [14]. Usually, physiological reflux begins between the ages of 1 to 6 months and improves progressively until the age of 12 months when it should resolve. If the symptoms start outside this age range or do not disappear around the age of 1, then the occurrence of GERD must be considered [5]. 

When it comes to pediatric patients, for children older than 8 years and adolescents, the same diagnostic criteria for GERD apply as for adults, since the typical symptoms are similar to theirs (i.e., heartburn, regurgitation). Nevertheless, in infants and small children differentiating between GER and GERD is more difficult, as the symptoms are varied and non-specific, among the most common being regurgitation, prolonged crying episodes, back arching, irritability (in Table 9 more symptoms are listed as well as signs by age groups) [15].

Also, in infants, the same constellation of symptoms can be found in cow’s milk protein allergy, making the two pathologies difficult to differentiate from a clinical point of view, especially when these symptoms do not correlate with the occurrence of reflux in esophageal pH-metry studies or do not remit after initiation of proton pump inhibitor treatment [16].

Regarding the epidemiological data, a systematic review on GERD in pediatric patients carried out in 2019, which included 25 eligible studies on the prevalence and characteristic symptoms of GERD, concluded that, in children aged 0 to 18 months, symptoms were present in more than one quarter of the cases, with their gradual decrease until disappearance until the age of 1. As for children over 18 months old, the percentage of those symptoms varied between 0 and 38% [17].

As mentioned above, anemia is recognized as one of the signs of GERD in children. The underlying mechanism by which it occurs is through corrosive esophagitis, and anemia might be the only presenting sign of this lesion due to GERD [14].

As stated in the beginning of the article, the effects of iron deficiency anemia in the human body are multiple, affecting several systems. On the short list, we note: in the central nervous system, it leads to mental and motor developmental delay, reduced cognitive function, irritability, and a shorter attention span. In the cardiovascular system, it can determine cardiac hypertrophy; in the immunologic system, it reduces the recovery rate after illnesses and increases the rate of respiratory infections, and decreases myeloperoxidase expression in leukocytes and the small intestine. At different cellular levels, it determines several perturbances such as ineffective erythropoiesis, increased auto hemolysis, decreased red cell survival, and oxidative damage to cell membranes [18].

It is known that there are multiple other causes of iron deficiency anemia, but for several, special attention must be paid. First of all, infants born prematurely have a faster growth rate that decreases the iron supply accumulated during pregnancy, but in premature births they are low already, because they are usually formed in the third trimester. Second of all, menstrual blood losses and, rarely, Von Willebrand disease are another source of IDA in adolescents; third of all, children with neuromotor pathology are an additional group predisposed to IDA due to needing gavage or a predominantly liquid diet that may be deficient in certain nutrients such as iron. Last, but not least, several gastrointestinal tract pathologies such as celiac disease, *Helicobacter pylori* infection, chronic inflammatory bowel disease intolerance to cow’s milk proteins, Meckel’s diverticulum, hiatal hernia, or parasitosis can lead to the path of insufficient iron absorption [6]. If gastroesophageal reflux adds to these particular situations, then the iron deficiency or IDA aggravates it.

The involvement of pediatric GERD in the pathophysiology of other entities such as asthma [19], recurrent pneumonia, recurrent wheezing [20], dental erosions [21,22], Sandifer syndrome [23], and sleep apnea [24] has been the subject of other adult or pediatric studies over time, but the relationship between iron deficiency anemia and gastroesophageal reflux disease, especially in children, was not the goal of many researchers. A query of PubMed Central electronic databases with the terms (“anaemia”[All Fields] OR “anemia”[MeSH Terms] OR “anemia”[All Fields]) AND (“gastroesophageal reflux”[MeSH Terms] OR (“gastroesophageal”[All Fields] AND “reflux”[All Fields]) OR “gastroesophageal reflux”[All Fields]) AND (“child”[MeSH Terms] OR “child”[All Fields] OR “children”[All Fields]) returned 1987 results, from which only a few, at a quick glance, contained the terms “Gastroesophageal reflux”, “anemia” or “iron deficiency” and “children” or “pediatric” united in the title [25,26,27,28]. In other articles relevant to the subject of our study, anemia was mentioned in the body of the text, as a sign of GERD or as a red flag for this pathology [1,4,5,29].

We identified studies that searched for causes of iron deficiency anemia unresponsive to treatment, among which GERD was counted as well. A retrospective study from 2006, conducted by a research group in Naples, Italy, found that 10 cases from a total of 238 studied had anemia due to blood losses determined by reflux esophagitis [30]. Another study published in 2007 in Cairo, Egypt determined the prevalence of celiac disease, *Helicobacter pylori,* and gastroesophageal reflux among a small group of 25 patients with iron deficiency anemia unresponsive to iron treatment received for 3 months. The research group concluded that gastroesophageal reflux was positive in 11 of the studied cases, but only 6 of them had gastroesophageal reflux solely, while the other 5 had an associated *H. pylori* infection or a combination of celiac disease and *H. pylori* infection in addition to GERD [25]. Another article published by a research group from the United States of America in 2022 described a series of five cases with iron deficiency anemia as a result of gastroesophageal reflux in children with congenital esophageal atresia or neurologic impairment. The article highlighted that these two conditions are at risk of developing GER and reflux can further lead to columnar metaplasia of the esophagus wall cells. This often leads to anemia even in the absence of gastrointestinal symptoms, suggesting that unexplained iron deficiency anemia in children with neurological disorders or esophageal atresia should be considered a consequence of gastroesophageal reflux and additional investigations would be useful in order to diagnose or rule out GERD [26].

A particular and infrequent form of gastroesophageal reflux disease seen typically in infants is Sandifer syndrome. This pathology associates certain movements and postures (spasmodic dystonia and opisthotonos) in the presence of reflux, it being considered that posturing is a way to alleviate pain caused by acidic reflux. Even though it represents less than 1% of pediatric cases of GERD, it must be taken into consideration as a form of gastrointestinal disorder and not be misinterpreted as a neurological disease in order to receive proper antiacid treatment [31]. Regarding the prevalence of anemia in this form of gastroesophageal reflux, we identified cases in the literature that are associated with anemia also or articles that pointed the presence of anemia as a complication of Sandifer syndrome [23,32,33].

Even if our study shows moderate correlation between GERD and anemia, there are still more patients that have associated anemia and GERD than those that have only anemia (23 versus 9). In addition, the results show an increased rate of iron deficiency among patients with GERD. Therefore, we consider it opportune to develop new studies in this direction, with larger groups, to evaluate the impact that this hematological pathology has on the pediatric population with gastroesophageal reflux disease.

## 5. Conclusions

Cumulating the hematological changes obtained in our study, we can say that GERD is accompanied by a degree of iron deficiency that opens the way to iron deficiency anemia. Considering the fact that anemia can be one of the signs of gastroesophageal reflux disease, especially among pediatric patients, this complication should not be neglected due to its long-term effects in a child’s development and further in adult life. When faced with a case of hypochromic hyposideremic anemia in which other causes have been excluded, the clinician should consider it as a result of a gastroesophageal reflux if the patient presents signs and symptoms suggestive for this gastrointestinal pathology, and also if the anemia does not respond to oral iron therapy. In addition, the small number of studies found in the literature regarding the prevalence of anemia in gastroesophageal reflux disease in children opens the road to further investigation.

## Figures and Tables

**Table 1:** Causes of iron deficiency anemia according to age and different pathologies [8].

**Age**	**Newborn**	Low birth weight
		Perinatal blood loss
		Low maternal iron stores
	**Infants, Small Children**	Excessive consumption of cow’s milk
		Inadequate dietary intake
		Intake of foods that disrupt iron absorption
	**Adolescents**	Growth spurt
		Menstrual period
		Von Willebrand disease
**Pathologies**	**Occult Bleeding**	Peptic ulcer
		Meckel’s diverticulum
		Intestinal polyp
		Hemangioma
		Inflammatory bowel disease
	**Insensible Blood Loss**	Celiac disease
		Chronic diarrhea
		Pulmonary hemosiderosis
		Parasitosis

**Table 2:** Normal hemoglobin and hematocrit values for age groups [11].

Age	Gender	Hemoglobin (g/dL)		Hematocrit (%)	
		IF	SL	IL	SL
**6 months up to 2 years**		11	13.5	31	42
**2–6 years**		11	13.7	34	44
**6–12 years**		11.2	14.5	35	44
**12–18 years**	**Girls**	11.4	14.7	36	46
	**Boys**	12.4	16.4	40	51

IL = inferior limit; SL = superior limit.

**Table 3:** Erythrocyte parameters values for age groups [11].

Age	MCV (fL)		MCH (pg)		MCHC (g/dL)	
	IL	SL	IL	SL	IL	SL
**0–1 years**	70.5	94.9	22.9	32.7	32.3	35
**1–3 years**	72.8	85.2	22.7	29		
**3–6 years**	77.4	89.9	25.2	29.3		
**6–13 years**			25.4	30.8		
**13–18 years**	77.6	95.7	25.9	32.4		

IL = inferior limit; SL = superior limit.

**Table 4:** Serum iron normal values for age groups [11].

Age	Serum Iron (mcg/dL)	
	IL	SL
**0–1 year**	20	153
**1–5 years**	9	151
**6–10 years**	6	148
**11–14 years**	19	156
**15–18 years**	14	156

IL = inferior limit; SL = superior limit.

**Table 5:** Statistical indicators for MCV in relation to GERD.

	Median MCV	Mean		Standard Deviation	Standard Error	Min	Max	Q25	Median	Q75
		−95%	+95%							
**With GERD**	77.02	75.82	78.23	7.66	0.61	56.25	102	74	77.89	82
**Without GERD**	80.75	78.89	82.62	7.02	0.93	61	92	77	81	86

**Table 6:** Statistical indicators for MCH in relation to GERD.

	Median MCH	Mean		Standard Deviation	Standard Error	Min	Max	Q25	Median	Q75
		−95%	+95%							
**With GERD**	25.94	25.5	26.37	2.74	0.22	17.29	33.8	24.8	26.35	27.5
**Without GERD**	27.2	26.56	27.83	2.38	0.32	19.36	31.1	26.07	27.6	28.5

**Table 7:** Statistical indicators for serum iron in relation to GERD.

	Median Serum Iron	Mean		Standard Deviation	Standard Error	Min	Max	Q25	Median	Q75
		−95%	+95%							
**With GERD**	58.93	50.82	67.04	27	4.02	15	131	35	54	77
**Without GERD**	73.37	51.5	95.24	36.19	10.04	1.78	143	52	66	100

**Table 8:** Distribution of cases according to gender and area of living.

	Gender		Area of Living	
	Boys	Girls	Urban	Rural
**With GERD**	92	80	110	62
**With anemia**	20	12	21	11
**With GERD and anemia**	14	9	15	8

**Table 9:** GERD signs and symptoms in children by age group [15].

Children Aged 1 to 5 Years	Children Aged 6 to 18 Years
Recurrent vomiting	Heartburn
Weight loss	Epigastric pain
Failure to thrive	Retrosternal pain
Refusal to feed	Nocturnal abdominal/retrosternal pain
Abdominal pain	Dysphagia
Difficulty swallowing	Nausea
Recurrent pneumonia	Nocturnal coughing
Anemia	Hoarseness
Chronic sinusitis or otitis	Sore throat
Dental erosions	Halitosis
Sleep disturbances, fatigue	Wheezing
Behavioral disorders, irritability	Recurrent pneumonia
	Chronic sinusitis or recurrent otitis media
	Laryngitis
	Dental erosions
	Sleep disturbances, irritability, behavioral disorder

## Data Availability

The data presented in this study are available on request from the corresponding author. The data are not publicly available due to ethical issues.
